# Deleterious Effects of Epicardial Adipose Tissue Volume on Global Longitudinal Strain in Patients With Preserved Left Ventricular Ejection Fraction

**DOI:** 10.3389/fcvm.2020.607825

**Published:** 2021-01-15

**Authors:** Gulinu Maimaituxun, Kenya Kusunose, Hirotsugu Yamada, Daiju Fukuda, Shusuke Yagi, Yuta Torii, Nao Yamada, Takeshi Soeki, Hiroaki Masuzaki, Masataka Sata, Michio Shimabukuro

**Affiliations:** ^1^Department of Diabetes, Endocrinology and Metabolism, School of Medicine, Fukushima Medical University, Fukushima, Japan; ^2^Department of Cardiovascular Medicine, Institute of Biomedical Sciences, Tokushima University Graduate School, Tokushima, Japan; ^3^Department of Community Medicine for Cardiology, Institute of Biomedical Sciences, Tokushima University Graduate School, Tokushima, Japan; ^4^Division of Endocrinology, Diabetes and Metabolism, Hematology, Rheumatology (Second Department of Internal Medicine), Graduate School of Medicine, University of the Ryukyus, Okinawa, Japan; ^5^Department of Cardio-Diabetes Medicine, Institute of Biomedical Sciences, Tokushima University Graduate School, Tokushima, Japan

**Keywords:** epicardial fat, global longitudinal strain (GLS), lipotoxicity, echocardiography, HFpEF (heart failure with preserved ejection fraction)

## Abstract

**Background:** It is known that epicardial adipose tissue (EAT) volume is linked to cardiac dysfunction. However, it is unclear whether EAT volume (EATV) is closely linked to abnormal LV strain. We examined the relationship between EATV and global longitudinal strain (GLS), global circumferential strain (GCS), and global radial strain (GRS) in patients with preserved LV function.

**Methods:** Notably, 180 consecutive subjects (68 ± 12 years; 53% men) underwent 320-slice multi-detector computed tomography coronary angiography and were segregated into coronary artery disease (CAD) (≥1 coronary artery branch stenosis ≥50%) and non-CAD groups. GLS, GCS, and GRS were evaluated by 2-dimensional speckle tracking in patients with preserved left ventricular (LV) ejection fraction (LVEF) ≥50%.

**Results:** First, GLS, but not GRS and GCS, was lower in the high EATV group though the LVEF was comparable to the low EATV group. Frequency of GLS ≤18 was higher in the high EATV group. Second, multiple regression model showed that EATV, age, male sex, and CAD, were determinants of GLS. Third, the cutoff points of EATV were comparable (~116–117 mL) in both groups. The cutoff of EATV ≥116 showed a significant correlation with GLS ≤18 in overall subjects.

**Conclusions:** Increasing EATV was independently associated with global longitudinal strain despite the preserved LVEF and lacking obstructive CAD. Our findings suggest an additional role of EAT on myocardial systolic function by impaired LV longitudinal strain.

## Introduction

The volume-based measurement of the left ventricular (LV) ejection fraction (LVEF) is a simple measure of the global systolic function that encompasses risk evaluation and the management of various cardiovascular diseases. However, this parameter is limited by pathophysiological entities where the ratio of the stroke volume to LV cavity size is preserved (1). This notion is well-applicable in the setting that patients with preserved EF (HFpEF) have a similar mortality rate to patients with reduced EF (HFrEF) (2, 3). A number of observational and interventional studies have validated the global strain of the LV myocardium measured by speckle tracking to be superior to both LVEF and LV filling parameters for predicting the outcome in HFpEF (4–6). Thus, adding global longitudinal strain (GLS), the most robust deformation marker, to LVEF increases the accuracy of predicting cardiovascular events in patients with ischemic or non-ischemic heart failure (4–6). GLS is often disturbed in preserved EF patients with hypertension (7), diabetes mellitus (8), LV hypertrophy (9), and cancer therapy-related cardiac dysfunction (CTRCD) (10, 11).

Poor GLS could be a marker of early cardiac dysfunction in obese individuals. There were reports that body mass index (BMI) (12–14) or visceral fat area (VFA) (15) was negatively associated with GLS. It has been suggested that the accumulation of epicardial adipose tissue (EAT) underlies cardiac dysfunction in obesity (16, 17). It is possible to hypothesize that the EAT volume (EATV) is more closely linked to an abnormal LV strain than other adiposity indices. However, studies evaluating this link are limited. Ng et al. showed that EATV is a determinant of LV strain independent of BMI and waist/hip ratio in patients without coronary artery disease (CAD) (18). LV strains can be affected by ischemic burdens in patients with preserved LVEF (1); therefore, the effects of CAD on LV strains are also to be elucidated.

In clinical imaging modalities, cardiac strain is represented by three principal directions (longitudinal, circumferential, circumferential, and radial) (19). LV myocardial fibers adjacent to the endocardium are longitudinally oriented and yield a longitudinal shortening; LV myocardial fibers in the middle layer are oriented circularly around the short axis and yield a radial shortening; and LV myocardial fibers adjacent to the epicardium are oriented obliquely and result in circumference shorting (19). Therefore, EATV might be linked differently to the strains of three layers. In this study, we examined the relationship between EATV and GLS, global circumferential strain (GCS), and the global radial strain (GRS) in CAD or non-CAD patients.

## Methods

### Study Population

We retrospectively analyzed 482 consecutive Japanese patients who had undergone cardiac computed tomography (CT) for the purpose of suspected CAD between 2012 and 2015 at Tokushima University Hospital (Supplement 1, Participant recruitment flow chart). Subjects were divided into the coronary artery disease (CAD, if ≥1 coronary artery branch stenosis of ≥50%) and non-CAD groups. The major exclusion criteria include serum creatinine levels >1.5 mg/dL; class III or IV heart failure; iodine-based allergy; acute coronary events, stroke, or coronary revascularization within the preceding 3 months; overt liver disease; hypothyroidism; and severe valvular disease. We had excluded acute coronary events because LV strain may be largely variable during course of acute coronary events. To exclude acute coronary events, we had evaluated medical records before and after cardiac CT and selected only chronic and stable CAD. Since the data for the validity of systolic function indices during atrial fibrillation (AF) are limited, we excluded AF patients. Additionally, we excluded patients either with LVEF <50% or regional LV wall motion abnormality to detect early systolic abnormalities of LV strains. Altogether, 180 patients were included in the full analysis set.

### Measurements

Trained staff measured the height, body weight, and blood pressure of the participants. Questionnaires were administered to record data on smoking history, use of anti-hypertensive drugs, anti-hyperglycemic drugs, and lipid-lowering drugs. A participant was recognized as having diabetes mellitus, when the fasting plasma glucose level was ≥126 mg/dL, or the HbA1c level was ≥6.5% (48 mmol/mol), or the participant was taking a regular medication of anti-hyperglycemic drugs. A participant was recognized as hypertensive, if systolic blood pressure was ≥140 mmHg, diastolic blood pressure was ≥90 mmHg, or if she/he was regularly taking antihypertensive drugs. A participant was recognized as having dyslipidemia, if the high-density lipoprotein (HDL)-cholesterol levels were <40 mg/dL (1.0 mmol/L), if low-density lipoprotein (LDL)-cholesterol levels were ≥140 mg/dL (3.6 mmol/L), or if triglyceride levels were ≥150 mg/dL (1.7 mmol/L), or if they were regularly taking lipid-lowering drugs.

### Quantification of Epicardial Fat Volume

Cardiac CT was performed using a 320-slice CT scanner (Aquilion One; Toshiba Medical Systems, Tokyo, Japan) having 0.275-ms rotation and 0.5/320/0.25 collimation (20). CT images were acquired using a retrospective, non-helical electrocardiogram-triggered acquisition mode protocol (tube voltage, 120 kV; tube current, 450 mA × 5 ms) with a thickness of 5-mm slices. All reconstructed CT image data were transferred to an offline workstation (Synapse Vincent, ver. 4.4, Fuji Film, Tokyo, Japan). EATV and local EAT thickness were measured as previously reported (17, 21, 22).

### Standard Echocardiographic Measurements

Echocardiography was performed using commercially available ultrasound diagnostic instruments in accordance with the guidelines issued by the American Society of Echocardiography (23). A complete 2D color, pulsed, and continuous-wave Doppler echocardiogram was performed. Imaging included apical two- and four-chamber views, from which LV and left atrial (LA) volumes were measured by the biplane method of disks using 2-dimensional images. The cavity dimension and wall thickness were measured in a parasternal long axis view. The left ventricular mass was estimated using the formula recommended by the guidelines. The measurement of the left ventricular ejection fraction was performed in biplane apical (2- and 4-chamber) views using a modified Simpson's method.

### 2-Dimensional Strain Echocardiography

The peak systolic LV strains were analyzed offline using a computer software program by EchoInsight software as described (24, 25). Briefly, the endocardium was automatically tracked throughout the cardiac cycle in the apical 4-chamber, 2-chamber, and long-axis views, after the manual definition of the LV endocardial border. Horizontal long-axis cines were tracked to derive longitudinal displacement and strain, while short-axis cines were used to derive the circumferential and radial displacements and strain. The strain values for the 6 basal, 6 mid, and 6 apical segments of the LV were averaged for GLS, GCS, and GRS (19). Yang et al. adopted cutoff values of GLS in surveillance of cancer chemotherapeutic-related cardiac dysfunction: >18% normal, 16–18% borderline and <16% is abnormal (10). Since our participants included non-CAD in addition to CAD subjects were considered to be normal to mild in GLS dysfunction, the value of GLS ≤18 was set as abnormal GLS (10).

### Statistical Analysis

The continuous and parametric values are expressed as mean ± standard deviation and categorical variables as percentage. The two-tailed unpaired student's *t*-test or chi-square test was used for group comparisons. Univariate or multivariate-adjusted regression analyses were performed to estimate the associations between potential determinants and longitudinal, radial, and circumference trains, the optimal cutoff values of EATV for predicting GLS ≤18 (10) were identified using receiver-operating characteristic (ROC) curves. Univariate or multivariate-adjusted odds ratios (OR) were also calculated to determine the clinical utility of the variables to estimate the GLS ≤18. Values of *P* < 0.05 were considered as statistically significant. Statistical analyses were conducted using the SPSS version 25 (SPSS, Inc., Chicago, Illinois, USA).

## Result

### General Characteristics

The general characteristics of patients with low EATV (<median EATV = 112 mL) and high EATV (≥median EATV = 112 mL) are shown in Table 1. EATV in the low and high EATV groups was 78 ± 25 and 162 ± 38 mL, respectively. In the high EATV group, the mean age and prevalence of male subjects were not statistically significant. There were no differences in the systolic and diastolic blood pressure and heart rate. The body weight and BMI were larger in the high EATV group. There was no difference between the total and LDL cholesterol; however, the HDL cholesterol was lower, and triglyceride was higher in the high EATV group. There was no difference between the fasting plasma glucose and HbA_1C_. Although smoking history and obesity were higher in the high EATV group, there was no difference in the proportion of hypertension, diabetes mellitus, or CAD.

**Table 1:** General characteristics of studied patients with low and high EATV.

**Parameters**	**Overall**	**Low EATV (<112 mL)**	**High EATV (≥112 mL)**	***P*-value**
Numbers	180	90	90	
Age (years)	68 ± 12	66 ± 12	70 ± 11	0.051
Male gender, *n* (%)	96 (53%)	42 (47%)	54 (60%)	0.060
Systolic blood pressure(mmHg)	134 ± 21	134 ± 24	134 ± 17	0.956
Diastolic blood pressure(mmHg)	75 ± 13	74 ± 13	76 ± 13	0.417
Heart rate (beats/min)	70 ± 12	71 ± 13	70 ± 11	0.867
**ANTHROPOMETRY**				
Body weight (kg)	63 ± 15	59 ± 15	68 ± 14	<0.001
Body mass index (Kg/m^2^)	25 ± 5	24 ± 5	26 ± 4	0.001
**BLOOD MEASUREMENTS**				
Total cholesterol (mg/dl)	196 ± 40	200 ± 40	191 ± 40	0.174
LDL cholesterol (mg/dl)	110 ± 32	111 ± 32	109 ± 32	0.788
HDL cholesterol (mg/dl)	61 ± 19	65 ± 20	58 ± 18	0.022
Triglycerides (mg/dl)	130 ± 66	116 ± 45	145 ± 82	0.008
Fasting plasma glucose (mg/dL)	120 ± 35	116 ± 35	125 ± 35	0.204
HbA1c (%)	6.1 ± 0.8	6.0 ± 0.9	6.1 ± 0.7	0.317
BNP (pmol/L)	49 ± 52	59.1 ± 61.5	38.4 ± 37.8	0.034
**MEDICATIONS**				
Antihypertensive medications, *n* (%)	82 (46%)	47 (52%)	35 (39%)	0.337
Lipid lowering medications, *n* (%)	45 (25%)	24 (27%)	21 (23%)	0.866
Antidiabetic medications, *n* (%)	35 (19%)	16 (18%)	19 (21%)	0.169
**COMORBIDITIES**				
Smoking history, *n* (%)	73 (41%)	27 (30%)	46 (51%)	0.003
Hyperlipidemia, *n* (%)	137 (76%)	67 (74%)	70 (78%)	0.507
Hypertension, *n* (%)	144 (80%)	71 (79%)	73 (81%)	0.597
Diabetes mellitus, *n* (%)	57 (32%)	25 (28%)	32 (36%)	0.262
Body mass index ≥25kg/m^2^, *n* (%)	94 (52%)	34 (38%)	60 (67%)	<0.001
Coronary artery disease, *n* (%)	112 (62%)	50 (56%)	62 (69%)	0.065
**EAT MEASUREMENTS**				
EATV (mL)	120 ± 53	77.6 ± 24.9	161.6 ± 37.5	<0.001
**LV MEASURES**			
Interventricular septum wall thickness (mm)	9.0 ± 2.0	9.2 ± 2.6	8.7 ± 1.1	0.119
LV posterior wall thickness (mm)	8.6 ± 1.5	8.6 ± 1.7	8.6 ± 1.1	0.763
LVDd (mm)	46.0 ± 5.0	45.1 ± 5.7	47.0 ± 4.0	0.015
LVDs (mm)	28.6 ± 4.8	28.2 ± 5.2	29.1 ± 4.2	0.204
LVEDV (mL)	85.2 ± 23.2	81.6 ± 22.3	88.8 ± 23.7	0.040
LVESV (mL)	29.9 ± 10.3	28.7 ± 9.9	31.2 ± 10.6	0.109
LVEF (%)	65.2 ± 5.1	65.3 ± 4.9	65.1 ± 5.3	0.793
LV mass index (g/m^2^)	83.9 ± 23.1	86.6 ± 25.7	81.3 ± 19.9	0.131
**LV STRAIN**				
GLS ≤18, GLS abnormality	53 (29%)	20 (22%)	33 (37%)	0.034
Global longitudinal strain (%)	−19.1 ± 1.3	−19.4 ± 1.2	−18.8 ± 1.4	0.003
Global circumferential strain (%)	−30.1 ± 6.2	−30.6 ± 6.4	−29.6 ± 6.0	0.322
Global radial strain (%)	27.2 ± 8.8	27.0 ± 8.3	27.4 ± 9.4	0.794

*Values are presented as the mean ± SD or n (%). Statistical significance were tested by independent t-test or chi-square test. n indicates of numbers of patients; EATV, epicardial adipose tissue volume; LVDd, left ventricular end-diastolic diameter; LVDs, left ventricular end-systolic diameter; LVEDV, left ventricular end-diastolic volume; LVESV, left ventricular end-systolic volume; LVEF: left ventricular ejection fraction; GLS, global longitudinal strain*.

**Figure 1:**
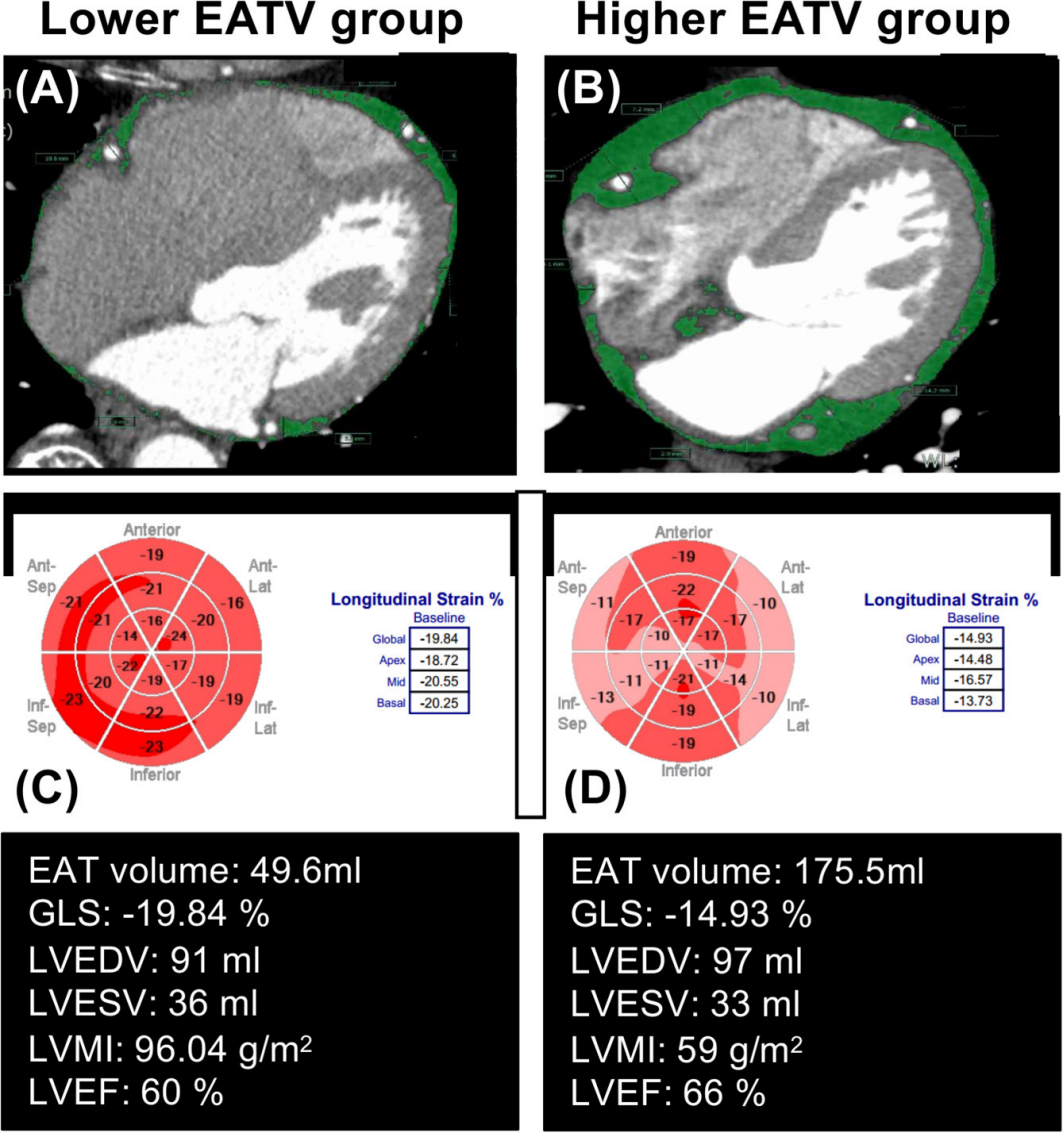
Representative measurements of global longitudinal strain (GLS), in patients with lower and higher epicardial adipose tissue volume (EATV). EAT measurements was manually placed **(A,B)** along the visceral pericardium; **(C,D)** the EAT area was automatically acquired as a density range between −190 and −30 HU in cardiac computed tomography. Compared with the lower EATV group **(A)**, with the group with higher EATV **(B)** had an impaired GLS (−19.84 vs. −14.93%), despite other clinical parameters being similar.

Among the echocardiographic parameters, LVDd and LVEDV were significantly greater in the high EATV group than in the lower EATV group and other measurements including LVEF and LV mass index were comparable between two groups. For LV strain, the global longitudinal strain (GLS) was lower in the high EATV group; however, the global radial strain (GRS) and global circumference strain (GCS) were comparable. The frequency of GLS ≤18 was higher in the high EATV group (37 vs. 22%, *P* = 0.034). Representative measurements of the global longitudinal strain (GLS) in patients with lower and higher EATV are shown in Figure 1.

### EATV and LV Strain

**Figure 2:**
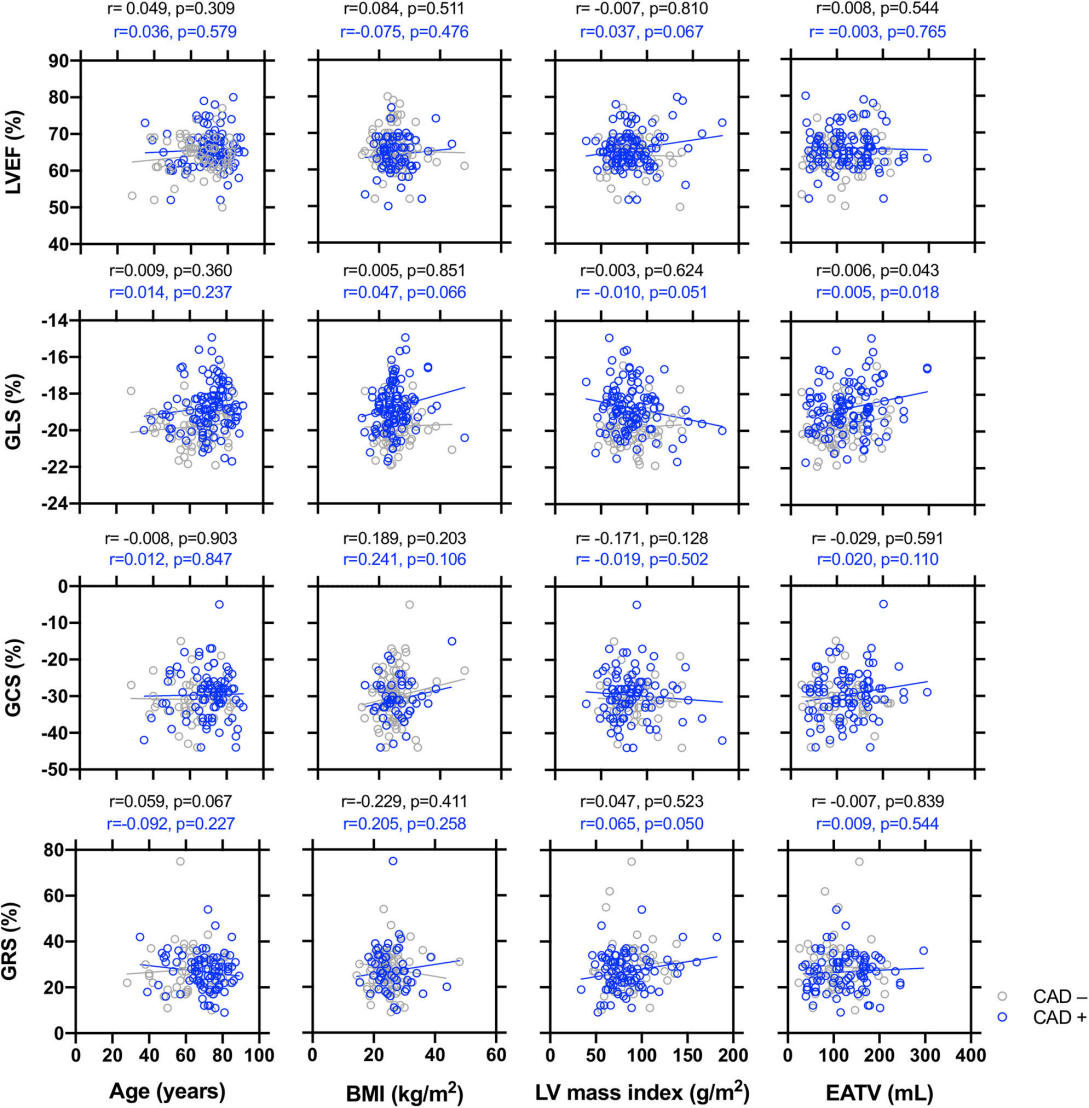
Simple correlations between the echocardiographic parameters of end systolic global longitudinal strain (GLS), global circumferential strain (GCS), and global radial strain (GRS), respectively with age, body mass index (BMI), left ventricular mass index (LVMI), and epicardial adipose tissue volume (EATV) in patients with or without coronary artery disease (CAD). A simple regression analysis was made separately in non-CAD (black circles and lines) and CAD patients (blue circles and lines). The *R* and *P*-values are shown.

The univariate regression analysis for the global longitudinal, radial, and circumferential strain in the overall group, non-CAD, and CAD patients is displayed in Supplement 2 and Figure 2. In the overall patient group: GLS was observed to decrease with age, male sex, the presence of CAD, and EATV, but increased with LVEF; GRS was observed to decrease with IVS, LVPW, and LVMI; GCS was observed to decrease with BMI, but increased with IVS and LVEF. In the non-CAD subgroup, GLS was observed to decrease with EATV, but increased with LVEF; GRS was observed to increase with LVPW; GCS was observed to increase with smoking history and LVEF. In the CAD subgroup, GLS was observed to decrease with the male sex and EATV, but increased with the LVEF; GRS was observed to increase with IVS; GCS was observed to increase with LVEF.

The relationship between the LVEF and LV global strains and age, BMI, LVMI, and EATV was plotted in Figure 2. LVEF and GCS did not correlate with age, BMI, LVMI, or EATV. GLS was observed to decrease with EATV in both the non-CAD and CAD groups. GRS decreased with LVMI only in the CAD group. In Supplement 3, the explanatory factors of global strain by multivariate analysis were examined. GLS decline was significantly associated with the male sex, CAD, and EATV (**Model 4**). GRS correlated only with hypertension (Model 4) and GCS correlated with BMI. Since EATV was associated only with GLS, we focused the subsequent analysis on the association between EATV and GLS.

### EATV Cutoff and OR for GLS Abnormality (GLS ≤18)

The general characteristics of patients with GLS > 18 and GLS ≤18 are shown in Table 2. The GLS ≤18 group was older and had higher prevalence of CAD. The body weight, BMI and blood pressure were comparable between two groups, while EATV was higher in the GLS ≤18 group. There was no difference in the fasting plasma glucose and HbA_1C_. Among the echocardiographic LV measures and LV strain, only GLS and GRS were significantly different between two groups.

**Table 2:** General characteristics of studied patients with GLS >18 and GLS ≤18.

**Parameters**	**GLS >18**	**GLS ≤18**	***P*-value**
Numbers	127	53	
Age (years)	66 ± 12	72 ± 10	0.008
Male gender, *n* (%)	64 (50%)	32 (60%)	0.175
Systolic blood pressure(mmHg)	135 ± 21	132 ± 20	0.390
Diastolic blood pressure(mmHg)	75 ± 14	74 ± 12	0.759
Heart rate (beats/min)	69 ± 12	72 ± 12	0.314
**ANTHROPOMETRY**			
Body weight (kg)	63.4 ±15.2	63.2 ±14.0	0.937
Body mass index (kg/m^2^)	25 ± 5	25 ± 4	0.906
**BLOOD MEASUREMENTS**			
Total cholesterol (mg/dl)	196 ± 38	195 ± 45	0.912
LDL cholesterol (mg/dl)	109 ± 30	111 ± 38	0.820
HDL cholesterol (mg/dl)	62 ± 19	61 ± 18	0.868
Triglycerides (mg/dl)	128 ± 66	134 ± 69	0.661
Fasting plasma glucose (mg/dL)	124 ± 38	110 ± 23	0.076
HbA1c (%)	6.1 ± 0.9	6.0 ± 0.8	0.774
BNP (pmol/L)	45 ± 51	60 ± 55	0.173
**MEDICATIONS**			
Antihypertensive medications, *n* (%)	64 (50%)	18 (34%)	0.352
Lipid lowering medications, *n* (%)	28 (22%)	17 (32%)	0.051
Antidiabetic medications, *n* (%)	27 (21%)	8 (15%)	0.846
**COMORBIDITIES**			
Smoking, *n* (%)	52 (41%)	21 (40%)	0.299
Hyperlipidemia, *n* (%)	94 (74%)	43 (81%)	0.139
Hypertension, *n* (%)	102 (80%)	42 (79%)	0.299
Diabetes mellitus, *n* (%)	46 (36%)	11 (21%)	0.042
Body mass index ≥25kg/m^2^, *n* (%)	64 (50%)	30 (57%)	0.375
Coronary artery disease, *n* (%)	67 (53%)	45 (85%)	<0.001
**EAT MEASUREMENTS**			
EATV (mL)	114 ± 49	133 ± 60	0.029
EATV median ≥112 mL yes or no	59 (47%)	33(67%)	0.053
EATV cutoff ≥116 mL yes or no	50 (39%)	33(62%)	0.005
**LV MEASURES**			
Interventricular septum wall thickness (mm)	9.1 ± 2.3	8.7 ± 0.9	0.244
LV posterior wall thickness (mm)	8.6 ± 1.6	8.5 ± 0.9	0.647
LVDd (mm)	46.2 ± 5.2	45.5 ± 4.4	0.373
LVDs (mm)	28.9 ± 4.7	28.0 ± 4.8	0.287
LVEDV (mL)	85.3 ± 23.2	84.9 ± 23.4	0.931
LVESV (mL)	29.6 ± 9.9	30.6 ± 11.3	0.570
LVEF (%)	65.5 ± 4.8	64.4 ± 5.6	0.199
LV mass index (g/m^2^)	85.7 ± 24.2	79.5 ± 19.6	0.111
**LV STRAIN**			
Global longitudinal strain (%)	−19.8 ± 0.8	−17.5 ± 0.8	<0.001
Global circumferential strain (%)	−31.1 ± 6.0	−27.9 ± 6.1	0.004
Global radial strain (%)	27.8 ± 8.8	25.8 ± 8.9	0.201

*Values are presented as the mean ± SD or n (%). Statistical significance were tested by independent t-test or chi-square test. n indicates of numbers of patients; EATV, epicardial adipose tissue volume; LVDd, left ventricular end-diastolic diameter; LVDs, left ventricular end-systolic diameter; LVEDV, left ventricular end-diastolic volume; LVESV, left ventricular end-systolic volume; LVEF: left ventricular ejection fraction; GLS, global longitudinal strain*.

**Figure 3:**
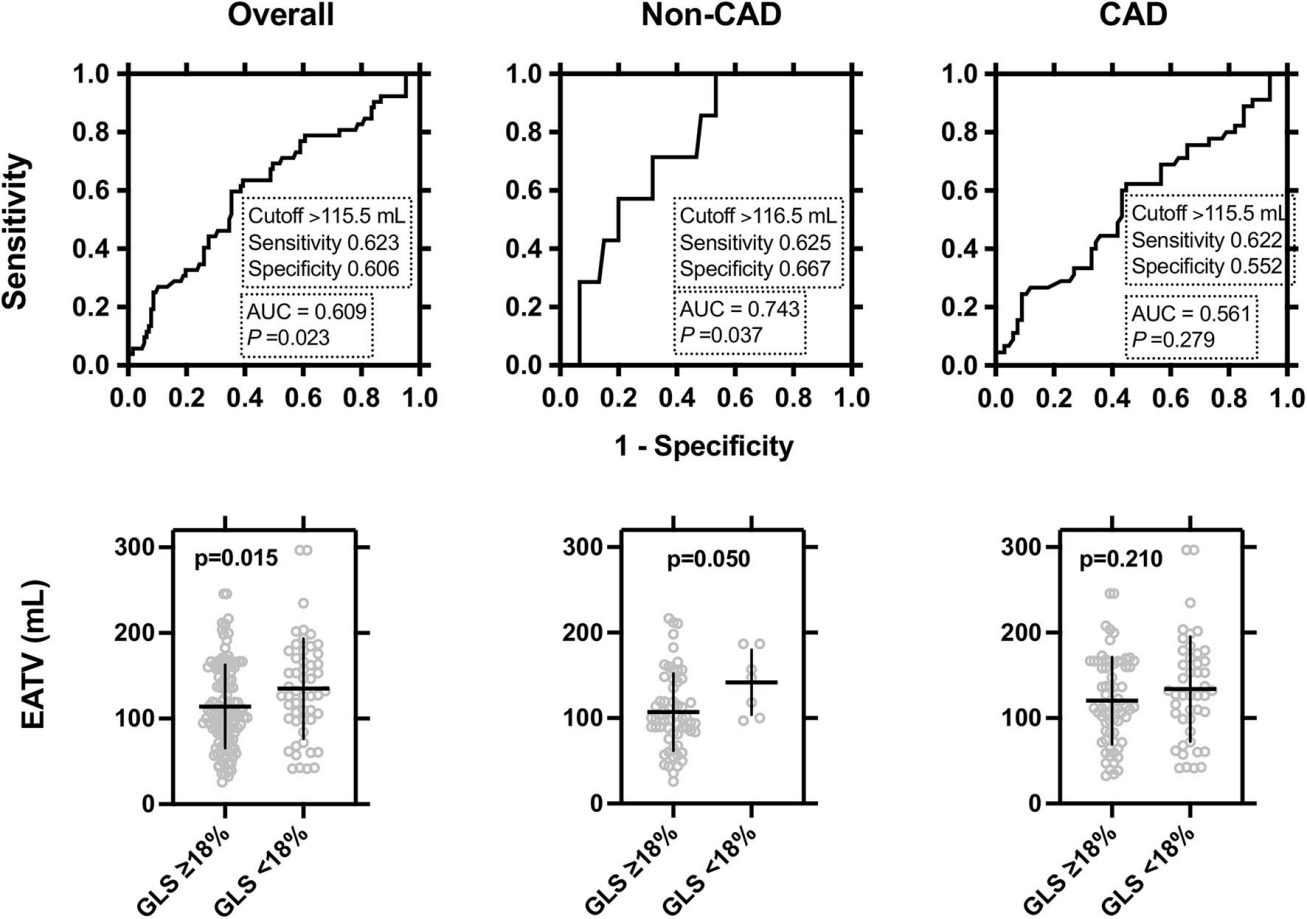
Receiver-operating-characteristic (ROC) curve analysis evaluating the predictive accuracy of the left ventricular (LV) global longitudinal strain in the overall, non-CAD, and CAD patients. Upper panel: The cut-off point of epicardial adipose tissue volume (EATV) for predicting GLS ≤18 and its sensitivity and specificity are shown. Lower panel: Comparisons of EATV between GLS ≤18 vs. GLS >18 were made by two-tailed unpaired t-tests and the statistical significance was set at *P* < 0.05.

The cutoff points of EATV for predicting GLS ≤18% in the ROC curve analysis are shown in Figure 3. The cutoff points were comparable among the overall (116 mL), non-CAD (117 mL), and CAD (116 mL) groups, respectively, and showed a significant power, in the overall and non-CAD groups, but not in the CAD group (Figure 3, upper panel). EATV was significantly higher in the GLS <18% group than in the patients with GLS >18% only in the overall study group and non-CAD groups (Figure 3, lower panel).

Finally, we calculated the ORs of the EATV cutoff for GLS ≤18%. As shown in Table 3, in the overall study group, EATV ≥116 was correlated with GLS ≤18 (crude OR 2.54 [95% CI 1.31–4.92], *P* = 0.006, and multivariate-adjusted OR 2.22 [1.03–4.79], *P* = 0.042) as well as diabetes mellitus and CAD. In the non-CAD and the CAD groups, EATV ≥116 mL did not reach to a significant correlation with GLS ≤18. In the CAD group, only diabetes mellitus was correlated with GLS ≤18 (crude OR 2.54 [95% CI 1.05–6.12], *P* = 0.038, and multivariate-adjusted OR 2.22 [1.04–6.94], *P* = 0.041).

**Table 3:** Logistic regression analysis to predict GLS abnormality (GLS ≤18).

	**Overall**				**non-CAD**				**CAD**			
**Variables**	**Crude OR (95%CI)**	***P***	Adjusted OR (95%CI)^*^	***P***	**Crude OR (95%CI)**	***P***	Adjusted OR (95%CI)^*^	***P***	**Crude OR (95%CI)**	***P***	Adjusted OR (95%CI)^*^	***P***
Age (per year)	1.04 (1.01–1.08)	0.010	1.03 (1.00–1.07)	0.094	1.05 (0.98–1.13)	0.172	1.02 (0.95–1.11)	0.555	1.03 (0.99–1.07)	0.200	1.04 (0.99–1.08)	0.106
Male sex (yes or no)	0.64 (0.33–1.23)	0.176	0.53 (0.22–1.29)	0.534	0.82 (0.19–3.58)	0.790	0.32 (0.05–2.17)	0.241	0.71 (0.32–1.54)	0.380	0.58 (0.20–1.62)	0.295
Body mass index (kg/m^2^)	1.00 (0.94–1.07)	0.906	1.03 (0.94–1.12)	0.544	0.93 (0.79–1.11)	0.440	0.86 (0.66–1.11)	0.243	1.03 (0.96–1.12)	0.407	1.06 (0.96–1.17)	0.241
Smoking history (yes or no)	1.02 (0.53–1.98)	0.945	1.82 (0.75–4.42)	0.186	3.77 (0.43–32.7)	0.229	6.44 (0.58–71.6)	0.130	1.03 (0.48–2.22)	0.933	1.35 (0.48–3.76)	0.567
Dyslipidemia (yes or no)	0.60 (0.26–1.36)	0.217	0.60 (0.22–1.60)	0.306	1.30 (0.28–5.99)	0.741	0.82 (0.14–4.81)	0.825	0.60 (0.21–1.70)	0.334	0.44 (0.13–1.53)	0.198
Hypertension (yes or no)	0.97 (0.43–2.20)	0.945	1.22 (0.47–3.16)	0.689	1.21 (0.22–6.69)	0.831	0.88 (0.11–6.84)	0.906	0.67 (0.27–1.63)	0.374	1.27 (0.42–3.85)	0.676
Type 2 diabetes mellitus (yes or no)	2.17 (1.02–4.62)	0.045	2.33 (1.01–5.38)	0.047	1.50 (0.28–8.11)	0.638	2.29 (0.28–18.9)	0.442	2.54 (1.05–6.12)	0.038	2.69 (1.04–6.94)	0.041
Coronary artery disease (yes or no)	5.04 (2.20–11.5)	<0.001	3.99 (1.65–9.79)	0.002	–	–	–	–	–	–	–	-
EATV ≥116 ml (yes or no)	2.54 (1.31–4.92)	0.006	2.22 (1.03–4.79)	0.042	3.33 (0.72–15.4)	0.123	6.29 (0.89–44.7)	0.066	2.03 (0.94–4.39)	0.072	1.61 (0.67–3.87)	0.285

*OR, odds ratio*.

* *OR adjusted for age, male sex, body mass index, smoking history, dyslipidemia, Hypertension, type 2 diabetes mellitus, coronary artery disease and EATV ≥116 ml. CAD, coronary artery disease; EATV, epicardial adipose tissue volume*.

## Discussion

In this study, we examined the relationship between the EAT and LV strains in patients with preserved LVEF. We obtained three major findings. First, the GLS, but not the GRS and GCS, was lower in the high EATV group though LVEF values were comparable to the low EATV group. The frequency of GLS ≤18 was higher in the high EATV group. Second, the multiple regression model showed that EATV as well as age, male sex, CAD, were determinants of GLS (Supplement 3, Model 4). Third, the cutoff points of EATV were comparable (~116–117 mL) in the overall, non-CAD, and the CAD groups. The cutoff of EATV ≥116 showed a significant correlation with GLS ≤18 in overall, but did not in the non-CAD and CAD groups. Taken together, our results demonstrated that EATV was a determinant of GLS abnormality in overall patients with preserved LVEF.

### EATV and General Characteristics

Although the LVEF values were comparable, the GLS, but not GRS and GCS, was altered in the high EATV group. Accordingly, the frequency of GLS ≤18 was higher in the high EATV group (37 vs. 22%, *P* = 0.034). Previous studies reported that patients with high EATV showed structural and functional alterations in the heart. Thus, high accumulations in EATV were reported to be correlated with a severity of CAD (21, 22, 26), progression of coronary high risk plaques (27), LV mass (16), and LV diastolic function (17). In contrast, the LVEF was not correlated with EATV (28) and patients with HFrEF would rather have a reduction in the EATV compared with normal controls (29). Collectively, the impact of EATV accumulation on cardiac indices can be various in patients' conditions and may be pronounced in preserved LVEF.

### EATV and LV Strain

The relationship between EATV and LV strains remains to be elucidated. Previous studies indicated that an increase in the BMI (12–14) was correlated with reduced GLS. Further, the current study clarified that EATV was a determinant of LV GLS independent of BMI in patients with preserved LVEF. This finding was consistent with the results of the report by Ng et al. (18). It has been shown that EATV was correlated with markers of LV mass (16) and LV diastolic function (E/e) (17), independent of BMI and VFA. Taken together, with respect to our results and Ng. et al. (18), the accumulation of EATV can be related to reduced GLS more strongly than whole body adiposity (12–14).

### EATV Cutoff and OR for GLS Abnormality (GLS ≤18)

For the first time, we evaluated the cutoff value of EATV for detecting global strain abnormalities and its diagnostic utility. The cutoff of EATV ≥116 mL showed a significant adjusted OR 2.22 [1.03–4.79] in overall subjects (Table 3). Reportedly, the cutoffs of EATV were 92 mL (CAD) (30), 100 mL (ACS) (31), 126 mL (cardiovascular events) (32), and 107 mL (high-risk plaque) (27), respectively. Our cutoff value of 116 mL is close to these values; therefore, it may share hidden cardiovascular risks. In the overall subjects, CAD and EATV ≥116 mL were significant determinants and diabetes mellitus was the sole determinant in the CAD group for GLS ≤18. EATV might affect GLS as well as the presence of CAD and diabetes mellitus.

### Potential Mechanisms

The mechanisms underlying the correlation between the accumulation of EATV and reduced GLS in patients with preserved LVEF remain to be elucidated. Three potential mechanisms were discussed below (33–35).

First, the accumulation of EATV may represent obesity-related systemic inflammatory disorders, which may promote cardiac dysfunction including GLS abnormality (33–35). Impaired LV global strain and/or heart failure (HFpEF) in obese individuals may be linked to systemic hemodynamic and hormonal abnormalities. It is noted that accumulated EATV closely related to visceral fat obesity (VFO) or central obesity, which is the potential risk of HFpEF (36, 37) through the development of diabetes mellitus, dyslipidemia, hypertension, and CAD (38). High EATV may be linked to reduced LV strains independent of the presence of diabetes, dyslipidemia, and hypertension as in VFO (33) via the activation of sympathetic nerve systems, production of reactive oxygen species (ROS) (39), chronic kidney disease (CKD), and proinflammatory immunometabolism (34). Moreover, our notion may be supported by the fact that increased EAT volume and insulin resistance were independently associated with increased myocardial fat accumulation and interstitial myocardial fibrosis (40).

Second, EAT may have local direct effects on the myocardium (33–35), which can modulate the LV strain. Hence, the accumulation of EATV may directly affect GLS via the paracrine action of EAT-derived cytokines. Notably, there are four components of lipids deposition in the heart: (1) circulatory and locally recruited fat, (2) intra- and extra-myocellular fat, (3) perivascular fat, and (4) pericardial fat, all of which are considered to modulate the LV strain via cellular cross-talk between the EAT and myocardium (lipotoxicity) (33, 41). Correlation of myocardial fat accumulation with GLS (40) supports this idea. Kramer et al. found that the subepicardial LV strain, as compared to the subendocardial strain, was largely impaired in the high-fat diet-induced obese model (42). A link between EAT accumulation and subepicardial strains might be suggested.

Third, EATV was associated with reduced GLS, but not with GRS and GCS, in the CAD and non-CAD groups. Haggerty et al. (43) demonstrated that EATV was negatively associated not only with GLS, but also with GCS and GRS. The reasons for this discrepancy could not be identified. Instead, our results agreed with Haggerty et al. (43) showing that the GRS was positively associated with the LV mass index (Figure 2). Previous studies showed that GRS was higher and GLS was lower in hypertensive patients with LV hypertrophy (44). It may be suggested that LV functional and structural remodeling, which can be affected independently by hypertension and EATV, may affect the GLS and GRS with different time courses. The current study showed that the GCS was negatively associated with BMI, but not with EATV (Supplement 2). This result agrees with a previous report (45). Theoretically, myocardial contraction can be classified according to the involved myocardial layer into (1) contraction of the subendocardial fibers contributing to longitudinal shortening, (2) contraction of the subepicardial fibers contributing to circumferential shortening, and (3) transmural fibers contributing to radial thickening (46). Studies in obese individuals reported that the GLS was commonly impaired; however, the changes in GRS and GCS were inconsistent between 2D and 3D (47–49), suggesting that this layer-specific strain measurements are being useful but still under clinical validation.

### Study Limitations

This study has potential limitations. First, the study design was cross-sectional, and it was conducted at a single center with a relatively small number of patients. Second, the patients consisted entirely of Japanese patients; therefore, the relevance of this study to other ethnic populations requires further research. Third, we did not consider the impact of patient medications or lifestyles on LV global strain. Fourth, we defined the impaired GLS as ≤18 based on the modification of Yang et al. (10), thereby limiting our results to moderate to severe GLS impairment. Fifth, previous studies show that accumulation of EATV is frequently linked to inflammatory status (26), suggesting that enhancement of chronic inflammation may underlie the link between EATV and GLS. However, because of the retrospective study design, we could not study the link in this study.

## Conclusion

This study found that increasing EATV is independently associated with the global longitudinal strain despite the preserved LVEF and lack of obstructive CAD. Our finding suggests the additional role of EAT on the myocardial systolic function by impaired LV longitudinal strain. The finding may help further our understanding of the link between obesity and heart failure with preserved LVEF.

## Data Availability Statement

The original contributions presented in the study are included in the article/Supplementary Materials, further inquiries can be directed to the corresponding author/s.

## Ethics Statement

The studies involving human participants were reviewed and approved by the Fukushima Medical University and Tokushima University ethics committees. Written informed consent was not provided because the study was done in a retrospective design.

## Author Contributions

MSh designed the research. GM collected data with the assistance of YT and NY. MSh and GM analyzed and interpreted data and wrote the manuscript with inputs from all other authors. KK and YT supervised the echocardiographic analysis. DF, SY, TS, HM, and MSa advised and discussed the study. MSh was the guarantor of this work, and as such, had full access to all the data in the study, takes responsibility for the integrity of the data, and the accuracy of the data analysis.

## Conflict of Interest

The authors declare that the research was conducted in the absence of any commercial or financial relationships that could be construed as a potential conflict of interest.
