# Supplementary figures and images for: Deleterious Effects of Epicardial Adipose Tissue Volume on Global Longitudinal Strain in Patients With Preserved Left Ventricular Ejection Fraction
Source: Front Cardiovasc Med. 2021 Jan 15;7:607825. doi: 10.3389/fcvm.2020.607825 (PMC7843424; doi:10.3389/fcvm.2020.607825)

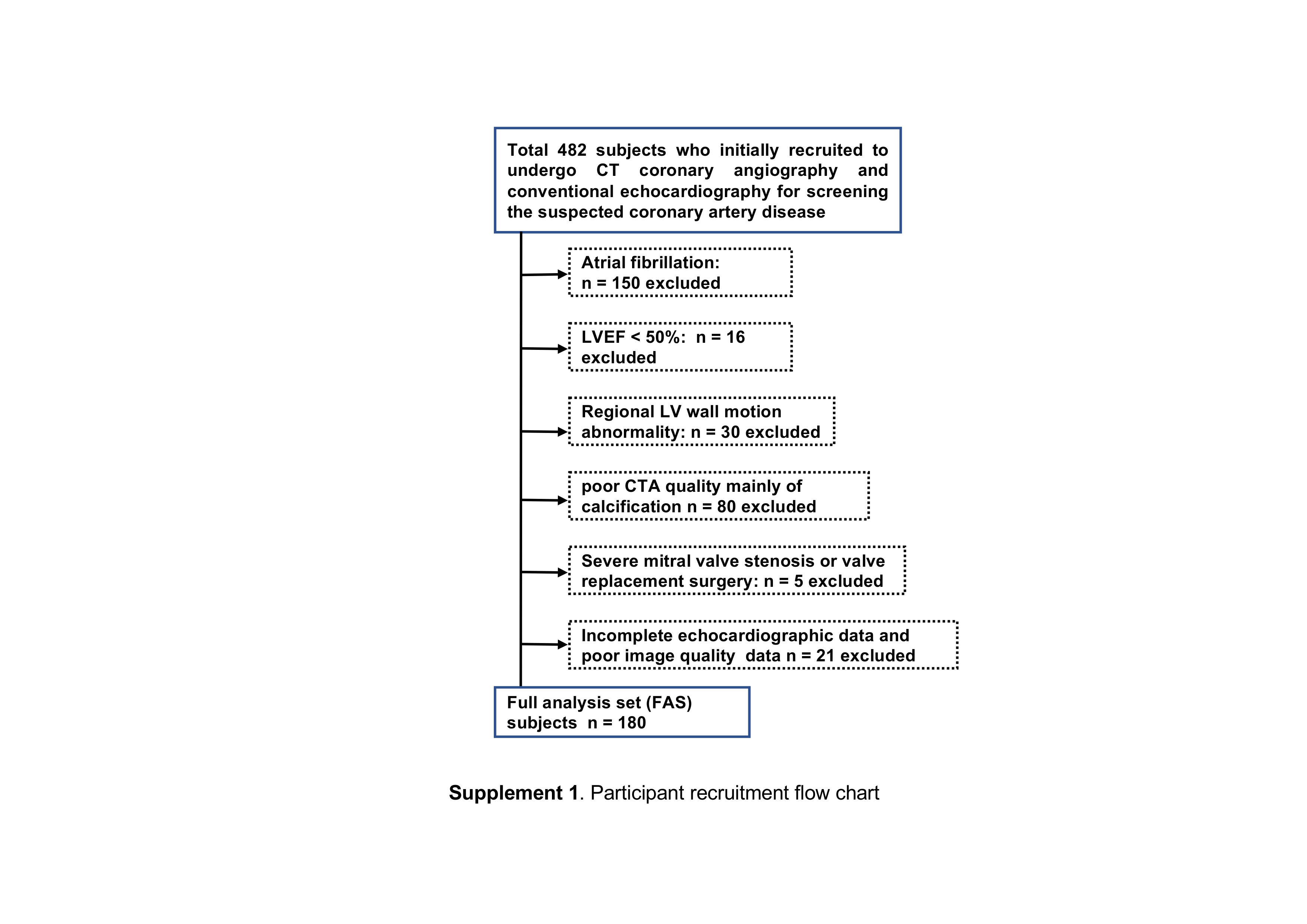

Supplement: Supplementary file 1 [file Image_1.TIF]

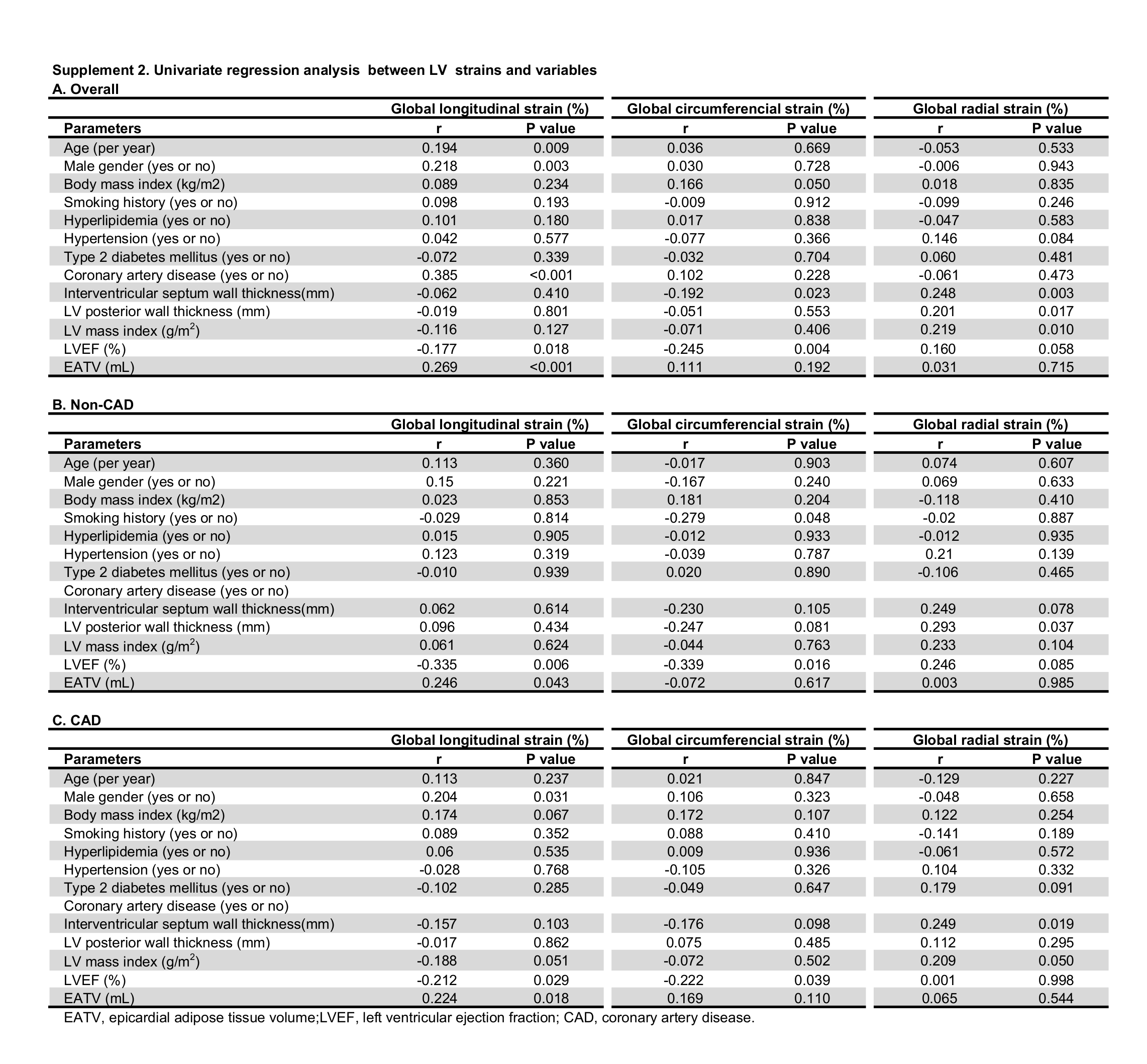

Supplement: Supplementary file 2 [file Image_2.TIF]

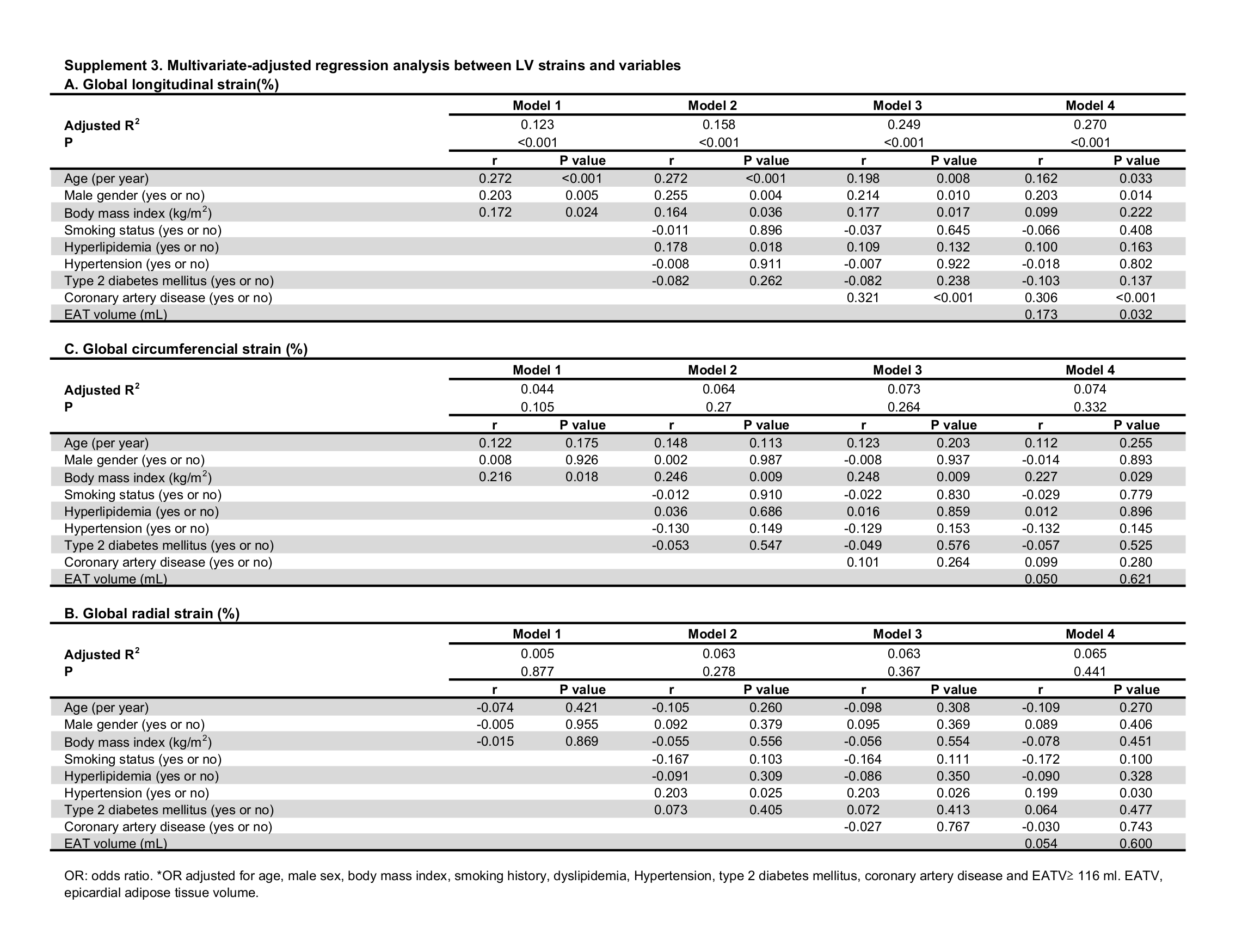

Supplement: Supplementary file 3 [file Image_3.TIF]
